# Single-Capacitor Electret Impact Microgenerator

**DOI:** 10.3390/mi7010005

**Published:** 2016-01-15

**Authors:** Igor Baginsky, Edward Kostsov, Alexey Sokolov

**Affiliations:** Institute of Automation and Electrometry, Russian Academy of Sciences, Siberian Branch, Koptug Prosp. 1, Novosibirsk 630090, Russia; kostsov@iae.nsk.su (E.K.); sokolovaa@iae.sbras.ru (A.S.)

**Keywords:** microgenerator, microvibrations, electrostatics, electret, impact

## Abstract

A new type of electrostatic microgenerator is presented that converts mechanical microvibrational energy into electric energy. The energy conversion mechanism is as follows. External microvibrations are transmitted to the device frame. The thin electret layer sputtered to the silicon substrate surface was fixed on the frame and the moving electrode was fixed by a weak suspension and comes into contact with the electret surface under the action of vibrations. A two-stage impact process is described: coming into contact with the spring stop that models the undulation of the contact surfaces, and direct impact on the electret surface. A numerical modeling of the generator operation is performed and analytic estimates of the generated power are obtained. It is shown that the energy generated by this motor is significantly higher than the energy generated by the classical mechanism based on the excitation of the forced vibrations of the moving plate. Experimental measurements of the microgenerator prototype parameters confirm the results of the theoretical modeling.

## 1. Introduction

Currently, the problem of powering remote microelectromechanical system (MEMS) devices calls for the development of direct current (DC) power sources with power ranging from 10 μW to 1 mW. One of the most promising power sources is the energy of surface microvibrations of solids [1]. Papers [2,3,4] analyzing different methods of microvibrations energy conversion into electric energy, electromagnetic, electrostatic and piezoelectric generators are described.

It was shown [5,6,7,8,9,10] that capacitive electrostatic microgenerators create the highest electric field energy density and, consequently, highest power [8,9,10,11]. In contrast to classical capacitive generators [4], electret generator [12] do not require an external power source; therefore, their design is simple and reliable, and they show the most promise for practical applications.

There are two types of capacitive microgenerators. In the first one, the electrodes of the capacitor perform the planar motion in their own plane (in-plane design) [13,14,15,16,17,18,19,20,21,22,23,24,25,26,27]. In the second one, the motion of the electrodes is normal to their plane, thus the distance between the electrodes changes (out-of-plane design) [16,28,29,30,31,32,33]. The force can be applied directly to the moving plate, e.g., in rotor generators [20], or it can be applied indirectly, as a result of mechanical–mechanical energy conversion in the mass-spring system.

The moving electrode (ME) motion depends on the applied force. In the out-of-plane design, the gap between dielectric (or electret) and ME surfaces (named “inter-electrode gap”) can range from 10–100 nm to a few microns, and high values of the energy density and specific electric power can be achieved [6,8,10]. In these generators, the maximum electric power is limited by the breakdown field strength in the inter-electrode gap, and with the operation frequency of 30–100 Hz it can be as high as 1–10 mW/cm^2^.

It should be noted that when microvibrations energy is used, it is almost impossible to apply a large force directly from the vibrations source to the moving electrode of the generator because it is impossible to affix the second electrode to a surface that is stationary with respect to the vibrating surface. Therefore, a two-stage process is required. In the first stage, mechanical–mechanical energy conversion takes place that converts part of the energy from the external vibrations source into the energy of the oscillatory circuit that consists of mass *m* and spring with the elasticity coefficient *k*. In the second stage, mechanical–electrical energy conversion is performed, where the energy of the oscillatory circuit is converted into the electric energy.

**Figure 1:**
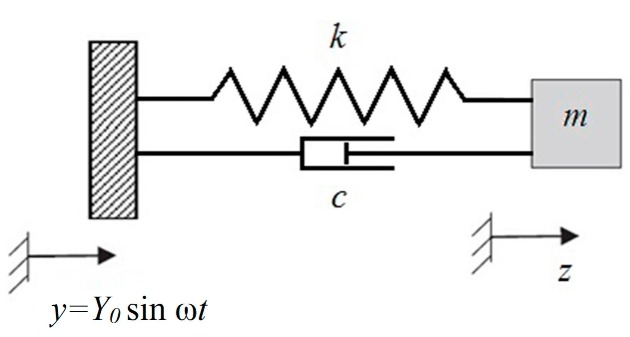
Mechanical scheme of vibrational energy harvesting. The generator frame is fixed on the vibration source *y*(*t*).

The spring is affixed to the generator frame that is placed on the surface that serves as the vibrations source. A moving electrode with the mass *m* is affixed to the other end of the spring (see Figure 1). Energy generation takes place when the distance *x*(*t*) between the frame and the mass *m* increases as the distance between capacitor plates increases due to capacitor plates moving against the forces of the electric field. The position of the frame with fixed electrode is given by *y*(*t*), and the position of the mass (or ME) is given by *z*(*t*), therefore the inter-electrode gap is *x*(*t*) = *z*(*t*) − *y*(*t*). In this microgenerator design, the mechanical forces acting against the electric field forces are the inertial force of the moving mass *m* that is equal to *ma* where *a* is acceleration of the moving electrode plate, and the elastic force of the spring, *kx*.

Only a small fraction of the energy of the vibrational source (which can be considered as an infinite source of energy) is passed into an oscillatory circuit since the value of *m* is limited by design. This can be considered the main drawback of this energy conversion mechanism. Thus, the maximum generated power is limited: in a certain position of the moving plate with respect to the attracting electrode, the inertial force passed onto the mass *m* via a spring becomes less than the electric field force, and the plate is irreversibly attracted to the electret surface. This sticking of the moving electrode leads to the stopping of the generator. To prevent electrode sticking, it is necessary to decrease the attracting force of the electric field, which limits the generator power.

In the planar design, this problem is solved by gradual decrease in the cross-area of the electrode surfaces as the capacitor plates separate, thus leading to the decrease in the counter-acting electric force. However, in the in-plane generators, it is impossible to use micron- and submicron inter-electrode gaps, since it is hard to ensure that ME and electret surfaces are parallel to a high degree of accuracy with the surface larger than 10–100 mm^2^. Therefore, the specific power of these generators is relatively low, on the order of 10 μW/cm^2^ (see, e.g., [18,19,20,21,22,23,24,25,26,27]), compared to the theoretical limit (see [26], where the generator power in the in-plane design with the force acting directly on the ME is shown to be as high as 200 μW/cm^2^).

Earlier we described an electrostatic energy microgenerator that has the following structure: stationary electrode–thin electret–moving electrode, where the energy conversion takes place as the moving electrode strikes the electret or stopper surface [34]. It can be noted that the surface impact mechanism is widely used in the commercially produced piezoelectric motors of the impact type that achieve power up to several tens of watts.

**Figure 2:**
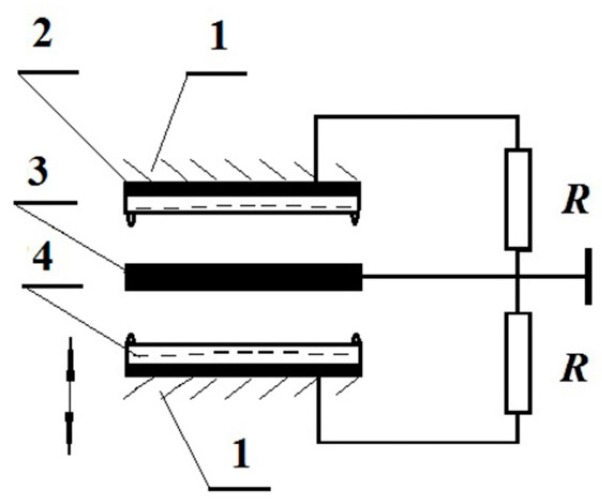
Two-capacitor impact generator: **1**—Housing; **2**—Electrode; **3**—Moving electrode; and **4**—Electret. Arrows show the direction of the housing vibrations.

To limit the vibrations amplitude of the ME that increases as the acceleration increases, the upper stopper is used. The system consists of the two capacitors that convert mechanical energy into the electric energy, which are connected through the moving electrode with the mass *m* (see Figure 2).

Compared to the classical electrostatic generator [13,14,15,16,17,18,19,20,21,22,23,24,25,26,27,28,29,30,31,32,33], under certain conditions in the impact mode, this generator was shown to generate power that is larger by a few orders of magnitude. In addition, one of the important advantages of the impact generator is its ability to convert vibrational energy from a wide frequency range.

The elastic deformation energy of the contact surfaces greatly affects the efficiency of the microgenerator operation. In the real structures, the contact surfaces are not perfectly flat: they can be convex or concave, thus the elastic forces with the large stiffness value can arise during mechanical contact.

Most papers analyzing operation of the capacitive generator do not take into account the effect of gravity on the ME motion. However, in many cases, the amplitudes of the vibroaccelerations are comparable to or less than the gravity acceleration g [1]; therefore, it is necessary to take into account the effect of the vibrating electrode weight on the energy generation.

This paper analyzes single capacitor electret out-of-plane impact generator taking into account the initial curvature of the contact surfaces and vibrating electrode weight.

## 2. The Structure and Mathematical Model

**Figure 3:**
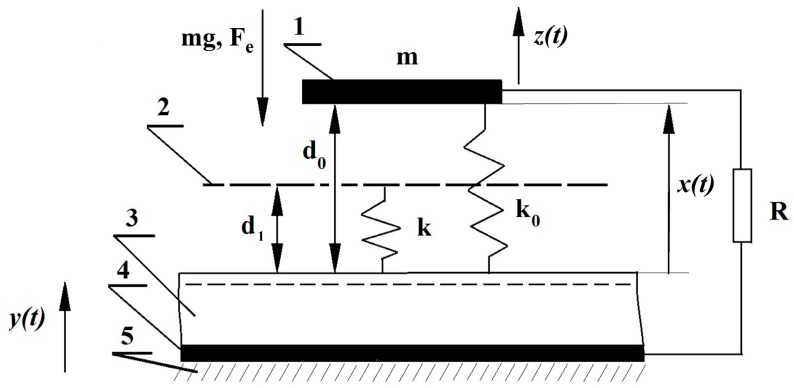
Schematic of the single-capacitor electret generator: **1**—Moving electrode; **2**—Moving stopper; **3**—Electret; **4**—Electrode; and **5**—Frame.

Mechanical and electrical schematic of the generator is shown in Figure 3. The curvature of the contact surfaces is roughly approximated by the stopper (shown as a dashed line) that is connected to the electret surface with the spring with the stiffness *k*.

In the initial contact of ME with the electret surface, the gap is equal to *d*_1_. The moving electrode with the mass *m* that is vibrating with respect to the electret surface is affixed to the generator frame by the suspension spring with the stiffness *k*_0_ << *k*. Besides, gravity force *P* = *m*g and electrostatic attraction act on the ME. Suppose gravity force *P* is much larger than suspension spring force but less than the elastic force of the stopper spring. Then the initial position of the moving electrode (in the absence of vibrations) is determined by the deflection ∆*x*_0_:

(1) $$\Delta x_{0}=(mg+F_{e})/k$$

where *F*_e_ is the electrostatic attraction force between the electrode and the electret surface:

(2) $$F_{e}=\frac{{Q_{s}}^{2}S}{2\varepsilon_{0}}$$

where *S* is ME area and *Q*_S_ is induced charge on the moving electrode that generally can be written as:

(3) $$Q_{S}(t)=-C(t)\left(V(t)+V_{P}\right)$$

where *V*(*t*) is the voltage on the structure (or on the load *R*) that is equal to zero in the initial state,

(4) $$V_{P}=\frac{Q_{P}}{C_{F}}$$

is electret characteristic potential, *Q*_P_ is electret surface charge density, *C*_F_ = εε_0_/*d* is specific capacity of the dielectric (electret) layer, ε is relative permittivity, ε_0_ is permittivity of free space and *d* is layer thickness.

The following equation describes time behavior of the device capacity and charge [6,8,10]:

(5) $$\frac{d}{dt}\left[C(t)\left(V(t)+V_{P}\right)\right]=-\frac{V(t)}{R}$$

When the moving electrode is caught at the spring stop *k*, the mechanical motion of the electrode follows the following expression:

(6) $$m\frac{d^{2}x}{dt^{2}}+c\frac{dx}{dt}+k(x-d_{1})-F_{e}-mg=-m\frac{d^{2}y}{dt^{2}}$$

where *y*(*t*) is the frame vibration, *x*(*t*) is the distance between the moving electrode and the electret surface and *c* is viscous friction coefficient. It can be shown that Equation (6) describes the system under the following assumption:

(7) $$a(t)\leq g+F_{e}(t)/m$$

where $a(t)=\frac{d^{2}y(t)}{dt^{2}}$.

It should be noted that if the pressing force (that is the sum of the electric field, gravity and inertial forces) is large, the spring force can be insufficient to hold ME above the electret surface. In this case, a quick contact between the ME and the electret surface takes place. Depending on the vibroacceleration value and mechanical losses during the impact the surfaces either stay in contact irreversibly (small acceleration, big losses) or separate, with the possibility of repeated impact.

Energy and momentum conservation lead to the following condition for the elastic impact:

(8) $${\frac{dx}{dt}|}_{-}={\frac{dx}{dt}|}_{+}$$

Indices “−“ and “+” denote relative velocity before and after the impact, respectively.

If Equation (7) is not satisfied, than for certain values of *a*(*t*) ME reaches the stopper *x* = *d*_1_, force of the spring *k* is insufficient, and ME moves above the stopper due to inertial forces, with the decelerating forces *P*, *F*_e_ and the suspension spring force *k*_0_(*d*_0_ − *x*) (where *d*_0_ is the height of the coil spring) acting on it. Here, the ME electrode motion is described as follows:

(9) $$m\frac{d^{2}x}{dt^{2}}-F_{e}-mg+k_{0}(d_{0}-x)=0$$

Even if *k*_0_ << *k*, the suspension spring force cannot be ignored, because for big enough displacement values *x* it can be significant.

## 3. Numerical Analysis

A system consisting of Equations (5), (6) and (9) with the condition in Equation (8) was solved numerically. As a result, the effect of the following structure parameters, the load resistance *R*; electret charge; stiffness of the suspension and stopper springs; ME mass; the distance between the stopper and the electret surface *d*_1_; and the external parameters, vibroacceleration *a*_0_ and vibration frequency *f*, on the generated power was determined.

The frame vibrations were assumed to follow the following equation:

*a*(*t*) = *a*_0_sin(ω*t*) (10)

**Figure 4:**
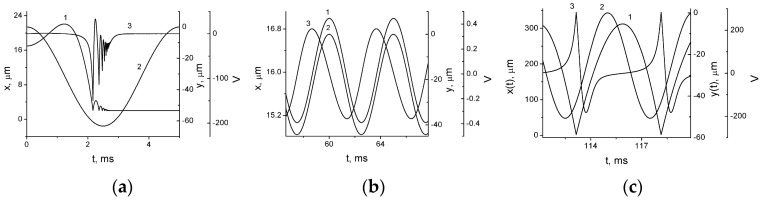
Kinetic curves of the excitation of electrode vibrations in the electret impact non-planar generator: (**a**) sticking mode: *a* = 50 m/s^2^, *k* = 5 × 10^4^ N/m; (**b**) forced vibrations mode: *a*_0_ = 35 m/s^2^, *k* = 2 × 10^5^ N/m; and (**c**) impact mode: *a* = 40 m/s^2^. *k* = 2 × 10^5^ N/m, *R* = 3 × 10^7^ Ω, *V*_P_ = −293 V. **1**—Inter-electrode distance *x*(*t*); **2**—Housing vibrations: External excitation *y*(*t*); **3**—Generated voltage. Parameters: *d*_1_ = *d*_0_ = 17 μm, *k*_0_ = 2 × 10^3^ N/m, *f* = 200 Hz, *R* = 3 × 10^5^ Ω, *S* = 1 cm^2^, *V*_P_ = +293 V.

We solved the system for *V*(*t*) values on the load. Then these values were used to calculate the generated electric power as a function of *R* for various external parameters, vibroacceleration amplitude and excitation frequency, and other system parameters shown in Figure 4.

Depending on the different values of the parameters, the following modes of operation can be observed:

1. Sticking mode: Irreversible electrostatic attraction of the ME to the electret surface. This mode occurs when the separation force that is the sum of the inertial force *ma* and spring force *kd*_1_ is insufficient to separate the surfaces. For a certain acceleration amplitude *a*_0_, after reaching a critical distance to the electret surface, ME is irreversibly attracted to the surface by the electrostatic forces, see Figure 4a. Note that in this mode the ME is not stuck initially to the electret surface, because electric force is not enough to overcome the spring force. When the inertial force *ma* grows more than the spring force *kd*_1_ ME and electret surfaces collide. As the distance *x* decreases, starting from some critical distance the electrostatic force becomes high enough to catch the ME. Therefore, after some impacts with growing frequency and decreasing amplitude during which the kinetic energy of ME is transformed into electrical energy in the load *R* the ME sticks to the electret surface.
2. Forced vibrations mode: In this case, ME that is initially in the equilibrium state with the distance to the electrode *x*(0) < *d*_1_ vibrates around this position without coming into contact with the electret surface. The vibrations are forced because the excitation frequency is much less than the natural frequency of the system.
3. Vibrational mode with the periodic impacts: Starting from certain *a*_0_ values, ME reaches the electret surface, the elastic impact takes place, ME separates from the surface, and, due to inertial forces *ma* the process is repeated, see Figure 4c. As shown in [34], the generated power reaches maximum values when one impact happens during one excitation period.

**Figure 5:**
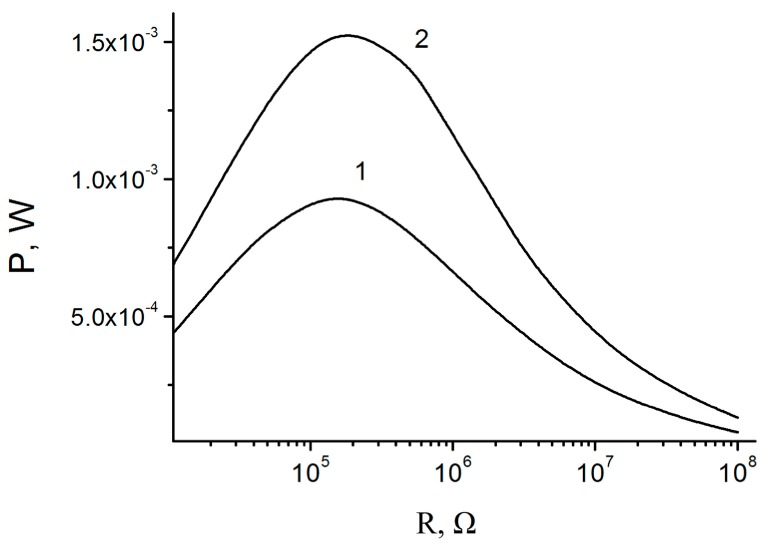
Load curves of the impact generator. *d*_1_ = *d*_0_ = 17 μm, *k* = 2 × 10^5^ N/m, *k*_0_ = 2 × 10^3^ N/m, *a*_0_ = 50 m/s^2^, *f* = 200 Hz, *S* = 1 cm^2^. *V*_P_ = 225 V (**1**), 293 V (**2**).

The analysis of the load parameters of the impact generator (Figure 5) shows that with *d*_1_ > 10 μm and stopper spring stiffness *k* ~ 10^5^–10^6^ N/m, even with high electret potential *V*_P_ ~ 200–300 V, the periodic impact mode can be achieved. The generator power can reach the values of 1–2 mW/cm^2^.

**Figure 6:**
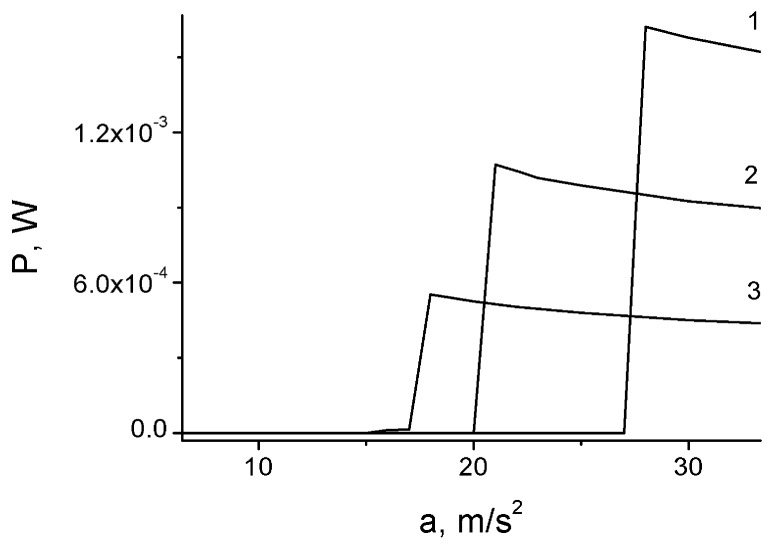
Output power of the generator as a function of external acceleration amplitude. *k* = 2 × 10^5^ N/m, *k*_0_ = 2 × 10^3^ N/m, *f* = 200 Hz, *R* = 3 × 10^5^ Ω, *d*_1_ = *d*_0_ = 17 μm, *S* = 1 cm^2^. *V*_P_ = 293 V (**1**), 225 V (**2**), 158 V (**3**).

A sharp increase (by many orders of magnitude) in the generated power can be achieved in the impact mode. This is demonstrated by a step on the plot of the power as a function of the vibrational acceleration amplitude (see Figure 6). As noted in [6,8,10], achieving high power in the capacitive generator (including electret generator) is contingent upon achieving high value of the capacity modulation depth in the vibrational mode: η = *C*_max_/*C*_min_ > 5. This value can be reached only in the impact mode, in other cases either η is small, or the surfaces cannot be separated.

Let us estimate mechanical power *P*_imp_ in the vibrator, as it determines the maximum electric power of the microgenerator. Since the frame vibrations are converted into kinetic energy of the ME motion upon impact, the mechanical power *P*_imp_ would be equal to the average kinetic energy generated during vibrations period of the frame ${\bar{W}}_{\text{imp}}$, multiplied by vibrations frequency *f*, where

(11) $${\bar{W}}_{\mathrm{imp}}=\frac{m}{2}\bar{v^{2}}=\frac{m}{4}{Y_{0}}^{2}\omega^{2}$$

*y*(*t*) = *Y*_0_sin(ω*t*) describes the motion of the frame, *Y*_0_ is vibrations amplitude, ω = 2π*f* is angular frequency. Then

(12) $$P_{\mathrm{imp}}={\bar{W}}_{\mathrm{imp}}f=\frac{m}{8\pi }{Y_{0}}^{2}\omega^{3}$$

For the vibrator as a mass-spring system, which in the general case operates in a forced vibrations mode, the mechanical energy generated in a period is estimated as [35]:

(13) $$W_{v}=ma_{0}\Delta x_{\max}=mY_{0}\omega^{2}\Delta x_{\max}$$

where *a*_0_ and Δ*x*_max_ are maximum vibroacceleration and ME displacement with respect to the frame. The ratio of the mechanical power of the impact and vibrational microgenerators is equal to:

(14) $$\frac{P_{\mathrm{imp}}}{P_{v}}=\frac{Y_{0}}{4\Delta x_{\max}}$$

where *P*_v_ = *W*_v_*f*.

If electric power of the generator is assumed to be proportional to the mechanical power, it is easy to conclude that for large enough vibrational displacement amplitudes the power of the impact generator is significantly higher than the power of the classical vibrational generator.

Therefore, everything else being equal, the use of impact generator leads to an output power that in some modes is by several orders of magnitude greater than the power of the classical vibrational generator, and can reach the value of order 1 mW/cm^2^. Published experimental values of the power reached in the traditional out-of-plane generators [27] typically do not exceed 10 μW/cm^2^.

**Figure 7:**
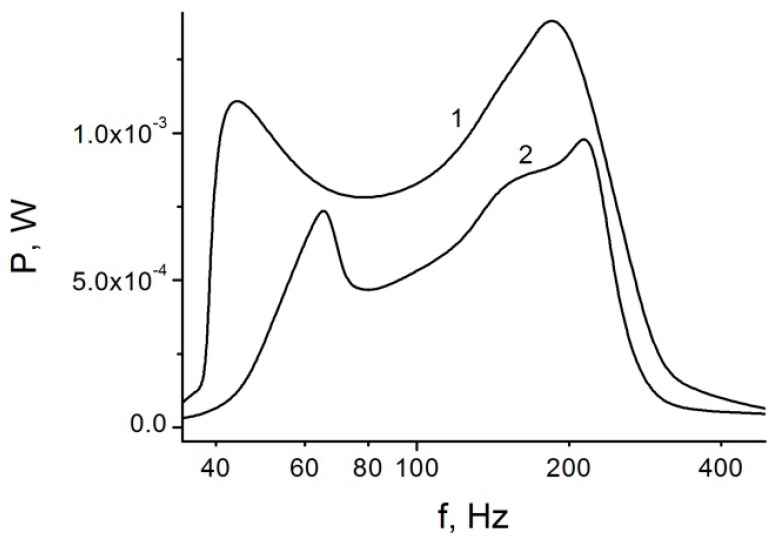
Generator power as a function of external vibrations frequency. *k* = 2 × 10^5^ N/m, *k*_0_ = 2 × 10^3^ N/m, *a*_0_ = 5 g, *R* = 3 × 10^5^ Ω, *d*_1_ = *d*_0_ = 17 μm, *S* = 1 cm^2^. *V*_P_ = 293 V (**1**), 225 V (**2**).

Frequency dependence of the generated power (Figure 7) shows two peaks that are attributed to the natural frequencies of the stop spring and coil spring. However, since those resonant vibrations are affected by the impacts, the peaks are observed at the lower frequencies compared to the natural vibrations frequencies, and they are smoother. The reason for the low power at the low and high frequencies is operation in the impactless mode. Because of relatively smooth dependence of power *vs.* frequency, this generator can utilize the vibrations energy in the wide range of frequencies compared to the traditional generators that use the resonance mode to increase output power [27,28].

## 4. Experimental

**Figure 8:**
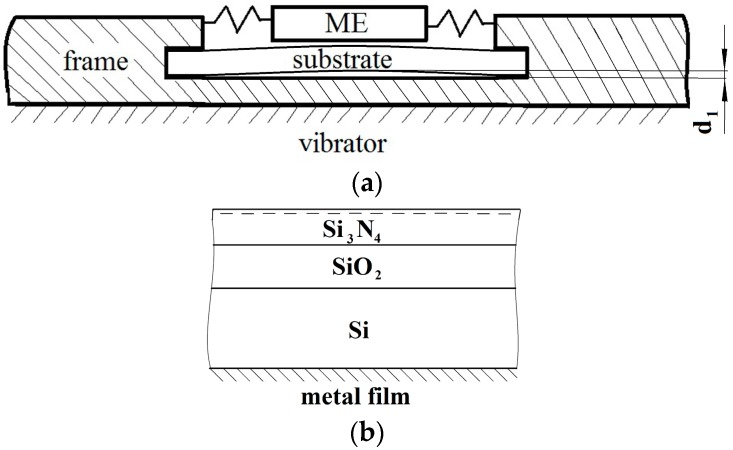
Impact microgenerator: (**a**) chematic picture; and (**b**) structure of electret film on the substrate.

The generator prototype (Figure 8a) consisted of the silicon substrate with the deposited electret layer, and the moving electrode. The substrate was affixed to the frame, and the moving electrode was pressed to the electret surface by the electrostatic force created by the electret charge and by the gravity force. The electret was created by sequential deposition of SiO_2_ and Si_3_N_4_ films. Pyrolitic deposition was used. The thicknesses of the films were 0.8 and 0.1 μm, respectively. To create ohmic contact, the aluminum film with 1 μm thickness was sputtered to the opposite side of the silicon substrate. Vacuum sputtering was used, followed by the vacuum annealing at 450 °C for 30 min. Negative charge was embedded to the silicon nitride surface using corona discharge technique [36,37]. The surface potential varied from −100 to −300 V, depending on the voltage applied to the metallic mesh above the film. The metallic wolfram needle with the curvature radius of 3–5 μm was used. The curvature was produced by the chemical etching. Then, 12 kV were applied to the needle, and the distance between the needle and the mesh was on the order of 5 cm.

The generator operation strongly depends on the geometry of the silicon substrate that was functioning as the spring *k* (see Figure 3). To achieve the necessary parameters, the substrate that had the shape of the rectangular plate was affixed from both sides. The substrate was somewhat bended, thus forming the gap *d*_1_ with the size of about 10 μm. Spring *k* operated in this gap (see Figure 8a). When the surfaces of the substrate and the housing came into contact as the silicon substrate was straightened due to the pressure from the moving electrode, an elastic impact occurred. It should be noted that when substrate was affixed to the housing along its whole perimeter, or when the whole substrate surface was affixed to the housing the generator power was significantly smaller due to the sticking effect. In this case generator operated only with the small *V*_P_ values, on the order of a few tens of volts.

As the external vibrations source, an electromagnetic device was used. The vibrations were measured using MEMS accelerometer ADXL203 (Analog Devices, Inc., Norwood, MA, USA) affixed to the vibrator housing.

**Figure 9:**
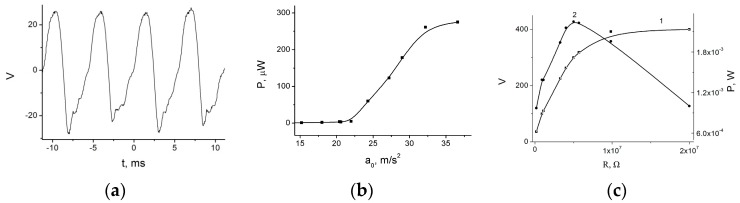
Experimental investigations of the impact out-of-plane electret generator: (**a**) generated voltage as a function of time in the impact mode (*V*_P_ = 110 V, *S* = 2.9 cm^2^, *a*_0_ = 22 m/s^2^, *R* = 10^7^ Ω); (**b**) output power as a function of the external vibrations acceleration amplitude (*V*_P_ = 110 V, *S* = 2.9 cm^2^, *R* = 10^7^ Ω); and (**c**) load curves of the generator: **1**—*V*(*t*), **2**—*P*(*t*) (*V*_P_ = 230 V, *S* = 6 cm^2^, *a*_0_ = 50 m/s^2^).

The voltage on the load *R* was measured as a function of time (see Figure 9a). In this case, the impacts correspond to the upper peaks on the *V*(*t*) curve. Right after the impacts, an abrupt change in voltage is observed, in this case towards negative voltage values. Voltage behavior on the load *R* qualitatively corresponds to the proposed model (see Figure 4a). Absence of the sharp voltage peak caused by impact is due to the differences between the spring *k* construction used in experiment and that assumed in the model. We will discuss these differences later. As the amplitude of the external vibrations acceleration grew, and the short impacts appeared, an abrupt growth of the generated power was observed (Figure 9b), in agreement with the proposed model. However, in this case, the growth is less pronounced. This growth leads to the strong growth of the generated power, which is reflected in the corresponding load curves (see Figure 9c). Power on the order of 0.3 mW/cm^2^ was generated with the moving electrode mass equal 25 g.

The experimental data qualitatively agree with the proposed model. In particular, sharp increase in the generated power was observed in the impact mode. It should be noted that the model only approximately describes the impact mechanism—The assumptions for the stopper spring *k* are substantially simplified. Therefore the predictions of the model do not agree quantitatively with the experimental results. In the experiment, the bent silicon substrate plate acted as the spring, thus causing the differences between the experimental results and model predictions. In addition, the model does not take into account the losses in the stopper spring *k* that occur during the impact of the surfaces during the experiment. Thus, experimental values of the generated power are lower than those predicted by the numeric analysis.

## 5. Discussion and Conclusions

Model of impact electret out-of-plane generator operation agrees qualitatively with experimental results. The following conclusions can be drawn:

1. The described generator design is simple and can be used to achieve high specific power, more than 1 mW/cm^2^.
2. The design with the two springs—suspension spring and stop spring—has a wide frequency band, and external vibrations in the wide frequency range can be used as a source of power, from several tens to several hundreds of hertz, which is the vibrations frequency range most often encountered in practice.
3. Impact operation mode is shown to depend on high external vibrations acceleration, more than 2–3 g, which means that the proposed design cannot be universally applied. However, these sources of vibrations can be readily found both in man-made and natural environments.
4. The discrepancy between the model and experimental data can be attributed to some inessential differences of the structures analyzed in the model and experiment. In the experiment, we have bent silicon substrate surface that serves as a stopper spring and some effective value of *d*_1_. There are also friction losses in the spring *k* and inelastic impact losses that are not taken into account in the model. Since the experimental generator geometry differs from modeling structure geometry, the process of impact is longer (see Figure 9a), resulting in slower growth of power at the transition to impact mode (Figure 9b) and smaller value of specific power (Figure 9c). However, this work aimed to show sharp increase in the generated power in the impact mode, and we have shown it both theoretically and experimentally.
